# Optimization of calcium carbonate precipitation during alpha-amylase enzyme-induced calcite precipitation (EICP)

**DOI:** 10.3389/fbioe.2023.1118993

**Published:** 2023-04-17

**Authors:** Norah Albenayyan, Mobeen Murtaza, Sulaiman A. Alarifi, Muhammad Shahzad Kamal, Abdulmohsen Humam, Manar M. AlAhmari, Amjad Khalil, Mohamed Mahmoud

**Affiliations:** ^1^ Department of Bioengineering, College of Chemicals and Materials, King Fahd University of Petroleum and Minerals, Dhahran, Saudi Arabia; ^2^ Center for Integrative Petroleum Research, King Fahd University of Petroleum and Minerals, Dhahran, Saudi Arabia; ^3^ Petroleum Engineering Department, King Fahd University of Petroleum and Minerals, Dhahran, Saudi Arabia; ^4^ Saudi Aramco, Dhahran, Saudi Arabia

**Keywords:** EICP, urea hydrolysis, alpha-amylase, soil stabilization, sand consolidation

## Abstract

The sand production during oil and gas extraction poses a severe challenge to the oil and gas companies as it causes erosion of pipelines and valves, damages the pumps, and ultimately decreases production. There are several solutions implemented to contain sand production including chemical and mechanical means. In recent times, extensive work has been done in geotechnical engineering on the application of enzyme-induced calcite precipitation (EICP) techniques for consolidating and increasing the shear strength of sandy soil. In this technique, calcite is precipitated in the loose sand through enzymatic activity to provide stiffness and strength to the loose sand. In this research, we investigated the process of EICP using a new enzyme named alpha-amylase. Different parameters were investigated to get the maximum calcite precipitation. The investigated parameters include enzyme concentration, enzyme volume, calcium chloride (CaCl_2_) concentration, temperature, the synergistic impact of magnesium chloride (MgCl_2_) and CaCl_2_, Xanthan Gum, and solution pH. The generated precipitate characteristics were evaluated using a variety of methods, including Thermogravimetric analysis (TGA), Fourier-transform infrared spectroscopy (FTIR), and X-ray diffraction (XRD). It was observed that the pH, temperature, and concentrations of salts significantly impact the precipitation. The precipitation was observed to be enzyme concentration-dependent and increase with an increase in enzyme concentration as long as a high salt concentration was available. Adding more volume of enzyme brought a slight change in precipitation% due to excessive enzymes with little or no substrate available. The optimum precipitation (87%) was yielded at 12 pH and with 2.5 g/L of Xanthan Gum as a stabilizer at a temperature of 75°C. The synergistic effect of both CaCl_2_ and MgCl_2_ yielded the highest CaCO_3_ precipitation (32.2%) at (0.6:0.4) molar ratio. The findings of this research exhibited the significant advantages and insights of alpha-amylase enzyme in EICP, enabling further investigation of two precipitation mechanisms (calcite precipitation and dolomite precipitation).

## 1 Introduction

Developing soil qualities has become unavoidable when it comes to identifying appropriate sites with substantial soil strength. Instead of observing regions with good geotechnical features, it is appropriate to apply soil stabilization techniques for site stability (Almajed et al., 2021). Soil stabilization increases the carrying capacity of the soil and reduces settlement and deformation. Soil stabilization is the most effective way to enhance ground strength (Wang et al., 2022). Several strategies have been explored by researchers (Afrin, 2017; Xue et al., 2021). The soil stabilization is performed through mechanical and chemical means (Firoozi et al., 2017; Ghadir & Ranjbar, 2018). The application of chemical soil stabilizers is on the rise and hundreds of chemicals are used for stabilization (Firoozi et al., 2017; Puppala and Pedarla, 2017; Mekonnen et al., 2020). Traditional ground or soil stabilization methods are costly, power intensive, and detrimental to the environment (Wang et al., 2023).

Biotechnology has acquired monumental attention and significance in the last few years. It is expanding its spans across many fields in boundless ways, and it is serving humanity through its applications in nutrition, food, medicines, and animal life sciences. It brings the advantage of living organisms and their goods to the enhancement of a sustainable environment and quality of human life (Verma et al., 2011). Biostabilization has lately emerged as a sustainable option capable of overcoming some of the limits of traditional soil improvement techniques (Umar et al., 2016; Rahman et al., 2020; Xue et al., 2023). Efforts are being made in soil stabilization by adapting bacteria to enhance soil performance (Banik et al., 2023; Sharma and Satyam, 2023). Enzyme-induced calcite precipitation (EICP) is a promising approach due to its simplicity of application in the field and compatibility across various types of soil (Saif et al., 2022). EICP in biofertilizers enhances soil geotechnical qualities by precipitating calcium carbonate (CaCO_3_), which adheres soil particles together (Kavazanjian and Hamdan, 2015). In addition to that, soil stabilization by microorganisms that precipitate CaCO_3_ is used in many soil types such as liquefiable soils, sand, sandy soil, and tropical residual soils; it is utilized for porous media remediation and the restoration of calcareous stone components (Xu et al., 2017; Zamani et al., 2021; Sharma et al., 2022). Winoto and Keawsawasvong used Sporosarcina pasteurii as a catalyst source for the reaction in the microbiologically induced calcite precipitation (MICP) and observed that the enzyme alone is sufficient for the required process to occur (Winoto and Keawsawasvong, 2022). EICP can also be used to seal rock fissures, clean wastewater, and minimize beach sand erosion (Xu et al., 2017). Furthermore, this bio-cementation technique is relatively recent and is used to improve soil quality. In EICP, calcium carbonate (CaCO_3_) precipitates out of the solution as a result of the hydrolysis of urea catalyzed by numerous enzymes (Hu et al., 2021). Soil reinforcement, pollutant remediation, and enhanced oil recovery by bio-plugging are just a few examples of the unique and sustainable engineering applications that stand to benefit greatly from EICP (Finnerty and Singer, 1983; Gowthaman et al., 2021; Omoregie et al., 2021; Sharma et al., 2021). It is vital to have a solid theoretical knowledge of the pace and amount of CaCO_3_ precipitation through the ureolytic chemical process for method improvement considering the vast number of alternative configurations for EICP (Ahenkorah et al., 2021b; Sharma et al., 2021). In a conventional EICP process, for example, the rate and amount of CaCO_3_ precipitation can be heavily influenced by the enzyme activity, concentration, and kinetic parameters of the enzymatic utilized, as well as the interaction conditions, such as pH and temperature (Kim et al., 2018).

It becomes indisputable that petroleum and other fossil fuels will continue to make up a sizable part of the global energy portfolio for the foreseeable future (Ismail et al., 2017). Oil production and processing facilities are permanently growing to keep up with the rising demand for energy throughout the world. Though the increase in production rates of oil and gas can be economical for the producer it brings a lot of challenges such as sand production. The sand production increases the drag force and *in situ* stress. It causes sand particles to move from reservoirs to the wellbore and come into the production stream. Most of the oil and gas reservoirs have poor sand consolidation and the companies are spending millions of dollars on sand production controlling technologies.

The petroleum industry has access to a wide range of methods for managing and controlling sand production. Among these methods are production rate control, mechanical ones like gravel packing or standalone screens, as well as chemical ones like resins (Weirich et al., 2013; Saghandali et al., 2022). Running the well at the maximum sand-free rate (MSFR) and performing effective sand management are another technique for limiting sand production (Stein et al., 1974; Rahim et al., 2010). In comparison to chemical methods, mechanical techniques are typically more expensive and time-consuming (ben Mahmud et al., 2020). They also face other issues like a decline in productivity index, complicated installation equipment, installation-related damage, workover difficulties, zonal isolation issues, collapsed screens, and equipment erosion and plugging (Arukhe et al., 2005; Abduljabbar et al., 2022). Additionally, they are useless at containing tiny sand particles. On the other hand, chemical methods are commonly used with a different strategy for managing the unconsolidated formation that generates a wide variety of sand. Since the beginning of 1940, sand production has been managed using chemical sand consolidation techniques, specifically plastics and resins (Parker et al., 1967). To create a bonding force between the loose sand grains, chemical sand consolidation methods involve injecting reactive chemicals into a target loose sand formation. With growing environmental concerns, there is a vital need for solutions that are economical, cost-effective, and environmentally friendly. Bio cementation techniques such as EICP is an effective tool to counter such environmental concerns. The EICP process can be an economical and viable means for sand consolidation to solve sand production issues in oil and gas wells (Alarifi et al., 2022). Calcium carbonate precipitated in the spaces as a result of chemical action, making the surface more compact and so enhancing the structural integrity. More than that, it is noteworthy that EICP has the potential to restrict CO_2_ leakage from a storage reservoir under a specific scope of temperature and pH (Phillips et al., 2013; Zamani et al., 2019).

Several enzymes have been used in Enzyme Induced Calcite Precipitation (EICP) for inducing the precipitation of calcium carbonate (CaCO_3_) from a supersaturated solution. These enzymes are typically calcium carbonate precipitation-inducing proteins, which are naturally occurring proteins found in various organisms that can induce the precipitation of calcite crystals (Polowczyk et al., 2016). Carbonic anhydrase is an enzyme that catalyzes the reversible hydration of carbon dioxide to form bicarbonate ions and protons (Noshi and Schubert, 2018; Molina-Fernández and Luis, 2021). Carbonic anhydrase can increase the concentration of bicarbonate ions in the solution, which promotes the formation of calcium carbonate crystals (Rodriguez-Navarro et al., 2019).

One of the most used enzymes in EICP is the urease enzyme (Hamdan and Kavazanjian, 2016; Yuan et al., 2016; Alarifi et al., 2022). This enzyme is abundant in microorganisms, plants, and some animals, particularly in legume seeds like jack beans. Urease facilitates the hydrolysis of urea into ammonia and carbon dioxide, which is crucial in the nitrogen cycle and has various industrial applications. The precipitation of calcite through enzymatic activity depends on factors such as enzyme type, reactivity, and purity. Therefore, the extraction and purification of enzymes should be cost-effective to make the EICP approach practical. The main source of urease used in commercial products is jack bean, which needs to be refined to achieve high-purity enzyme. However, performing EICP using urease enzymes on a large scale may be too expensive, making it necessary to find alternative sources of the enzymes. One possible alternative to jack bean urease is the amylase family enzymes which are plentifully available and cost effective alternate.

The amylase enzymes are one of the main enzymes that are widely employed in the industry which is a great incentive to utilize it for the petroleum industry. There are several different types of amylase enzymes, including alpha-amylase, beta-amylase, and glucoamylase, which differ in their specificities and mechanisms of action. Alpha-amylases account for 33% of the global enzyme output (Hu and Liu, 2021). The variety and magnitude of its applications make it a highly indispensable enzyme (Farooq et al., 2021). One exciting potential use of alpha-amylase is for sand stabilization in the oil and gas industry. However, before it can become a reality, extensive laboratory testing is required to confirm its suitability. This testing should investigate factors such as precipitation product, pH effect, temperature effect, and salinity effect to ensure the enzyme can be effectively applied in oil and gas operations. By harnessing the power of alpha-amylase, the petroleum industry could benefit from improved sand stabilization and other applications.

These enzymes’ main role is to hydrolyze starch molecules into polymers made up of glucose units. Amylases have the potential to be used in a broad range of commercial processes, including those in the food, fermentation, and pharmaceutical sectors. Animals, plants, and microbes may all produce amylases. Nevertheless, enzymes derived from fungi and bacteria have predominated in industrial applications (de Souza and e Magalhães, 2010). *Bacillus subtilis* is a well-known alpha-amylase producer (Raul et al., 2014; Lahiri et al., 2021). The activity of alpha-amylase refers to the ability of the enzyme to break down long-chain carbohydrates, such as starch, into smaller molecules like maltose, glucose and dextrin. This activity is measured in a laboratory setting using a variety of methods, including colorimetric assays, iodine-starch test and gel electrophoresis.

In this study, we investigated the process of EICP (Enzyme-Induced Calcium Carbonate Precipitation) using a new enzyme called alpha-amylase. The objective of the study is to optimize the parameters involved in EICP to achieve maximum calcite precipitation. The study examines several parameters that affect the process of EICP, including enzyme concentration, enzyme volume, the concentration of calcium chloride (CaCl_2_), temperature, the synergistic impact of magnesium chloride (MgCl_2_) and CaCl_2_, the concentration of xanthan gum, and solution pH. By studying these parameters, optimal conditions for EICP involving alpha-amylase as enzyme, can be identified. The output of this study could be valuable in a variety of applications, such as biomineralization, soil stabilization, and remediation of contaminated soils in the geotechnical as well as in the oil and gas industry.

## 2 Materials and methods

In this study, precipitation tests were conducted to investigate the activity of enzyme and precipitation using a novel enzyme, Alpha-amylase. The chemicals utilized in the study are urea, calcium chloride, magnesium chloride, potassium hydroxide, and xanthan gum which were acquired from Sigma Aldrich. The Alpha-amylase was supplied by MN-CHEM Saudi Arabia.

In this test, the reactant and enzyme were prepared in different test tubes and mixed later. The tests were conducted in closed tubes without exposure to the air. The tests were conducted at different concentrations of reagents and enzymes and the best optimum concentrations were selected for detailed investigations. The enzymatic reaction and precipitation were observed for 48 h. At the end of the reaction time, precipitations were measured by weight%.

Upon finding the optimum recipe, the further impact of different parameters was investigated on precipitation. The studied parameters are enzyme concentration, enzyme volume, the effect of MgCl_2_, xanthan gum concentration, pH, and temperature.

### 2.1 Test tube test

To measure the influence of initial chemical conditions on EICP and its reactivity, precipitation tests were carried out in test tubes. The precipitation was impacted by different parameters and their impact on precipitation was inspected by changing different variables including the impact of preliminary urea and CaCl_2_ concentrations, synergistic impact of MgCl_2_ and CaCl_2_, the effect of alpha-amylase enzyme volume and concentration, the initial composition of reactants, the effect of pH, and the effect of temperature. The testing conditions and formulations test are mentioned in detail in Tables 1–4.

**TABLE 1:** The chemical formulation used for the enzyme concentration sensitivity in presence of CaCl_2_.

Urea (mol)	CaCl_2_ (mol)	Enzyme (wt%)
1	1	5% 10% 15%
1	1.5	5% 10% 15%
1	2	5% 10% 15%

**TABLE 2:** Chemical formulation to estimate the synergistic effect of MgCl_2_ and CaCl_2_ at different concentrations of enzymes.

Urea (mol)	CaCl_2_ (mol)	MgCl_2_ (mol)	Enzyme (wt%)
1	0.9	0.1	5 10 15
1	0.8	0.2	5 10 15
1	0.7	0.3	5 10 15
1	0.6	0.4	5 10 15
1	0.5	0.5	5 10 15
1	0.4	0.6	5 10 15
1	0.3	0.7	5 10 15
1	0.2	0.8	5 10 15
1	0.1	0.9	5 10 15

**TABLE 3:** Chemical formulations used for the effect of the enzyme volume sensitivity.

Urea (mol)	CaCl_2_ (mol)	MgCl_2_ (mol)	Enzyme (wt%)	EICP volume (mL)	Enzyme volume (mL)
1	0.6	0.4	15	25	5
1	0.6	0.4	15	25	10
1	0.6	0.4	15	25	15
1	0.6	0.4	15	25	20
1	0.6	0.4	15	25	25
1	0.6	0.4	15	25	30

**TABLE 4:** Chemical formulations used for the effect of Xanthan Gum and temperature sensitivity.

Urea (mol)	CaCl_2_ (mol)	MgCl_2_ (mol)	Enzyme (wt%)	Xanthan gum (g/L)	Temperature °C
1	0.6	0.4	15	1	25
1	0.6	0.4	15	1.5	25
1	0.6	0.4	15	2	25
1	0.6	0.4	15	2.5	25
1	0.6	0.4	15	3	25
1	0.6	0.4	15	1	50
1	0.6	0.4	15	1.5	50
1	0.6	0.4	15	2	50
1	0.6	0.4	15	2.5	50
1	0.6	0.4	15	3	50
1	0.6	0.4	15	1	75
1	0.6	0.4	15	1.5	75
1	0.6	0.4	15	2	75
1	0.6	0.4	15	2.5	75
1	0.6	0.4	15	3	75
1	0.6	0.4	15	1	100
1	0.6	0.4	15	1.5	100
1	0.6	0.4	15	2	100
1	0.6	0.4	15	2.5	100
1	0.6	0.4	15	3	100

pH effect on precipitation was investigated by changing the pH by adding different concentrations of KOH in the base sample composed of (1 M urea, 0.6 M CaCl_2_, 0.4 M MgCl_2,_ and 15% enzymes). The added concentrations of KOH are (0.25, 0.5, 0.75 & 1) % molar. Initially, the temperature used for all the tests is 25°C then the effect of temperature on the activity of the enzyme was investigated by varying the temperature (25, 50, 75 & 100)°C (Table 4).

To conduct the test tube experiment, solutions of urea, salts, and catalyst were first prepared separately in accordance with the required concentrations listed in Tables 1–4. The solutions were then combined according to their mixing percentages and left to catalyze the precipitation reaction in a test tube at room temperature for 48 h, based on prior literature reports (Soon et al., 2014). After the 48-h period, the resulting fluid was filtered out and the reaction product that had accumulated in the test tube and on the filter paper was dried for 24 h in an oven at 70°C (Tariq et al., 2022). Once dried, the total weight of the reaction product was measured, excluding the weight of the test tube and filter paper. The total weight includes CaCO_3_ and other reaction products. By measuring precipitation proportion, the mass of CaCO_3_ formed was estimated (Eqs 1, 2) (Putra et al., 2017a):

Precipitation calculation equation:

$$Precipitation\;ratio\;\left(\%\right)=\frac{Actual\;mass\;of\;the\;precipitate}{Theoretical\;mass\;of\;CaCO_{3}}$$ (1)

$$Theoretical\;mass\;of\;{CaCO}_{3}=C\times M\times V$$ (2)

Where;

C: total concentration of the solution (M)
M: CaCO_3_ molar mass (100.087 g/mol)
V: total volume in the test tube (mL)

### 2.2 Compositional and microstructural analysis

**FIGURE 1:**
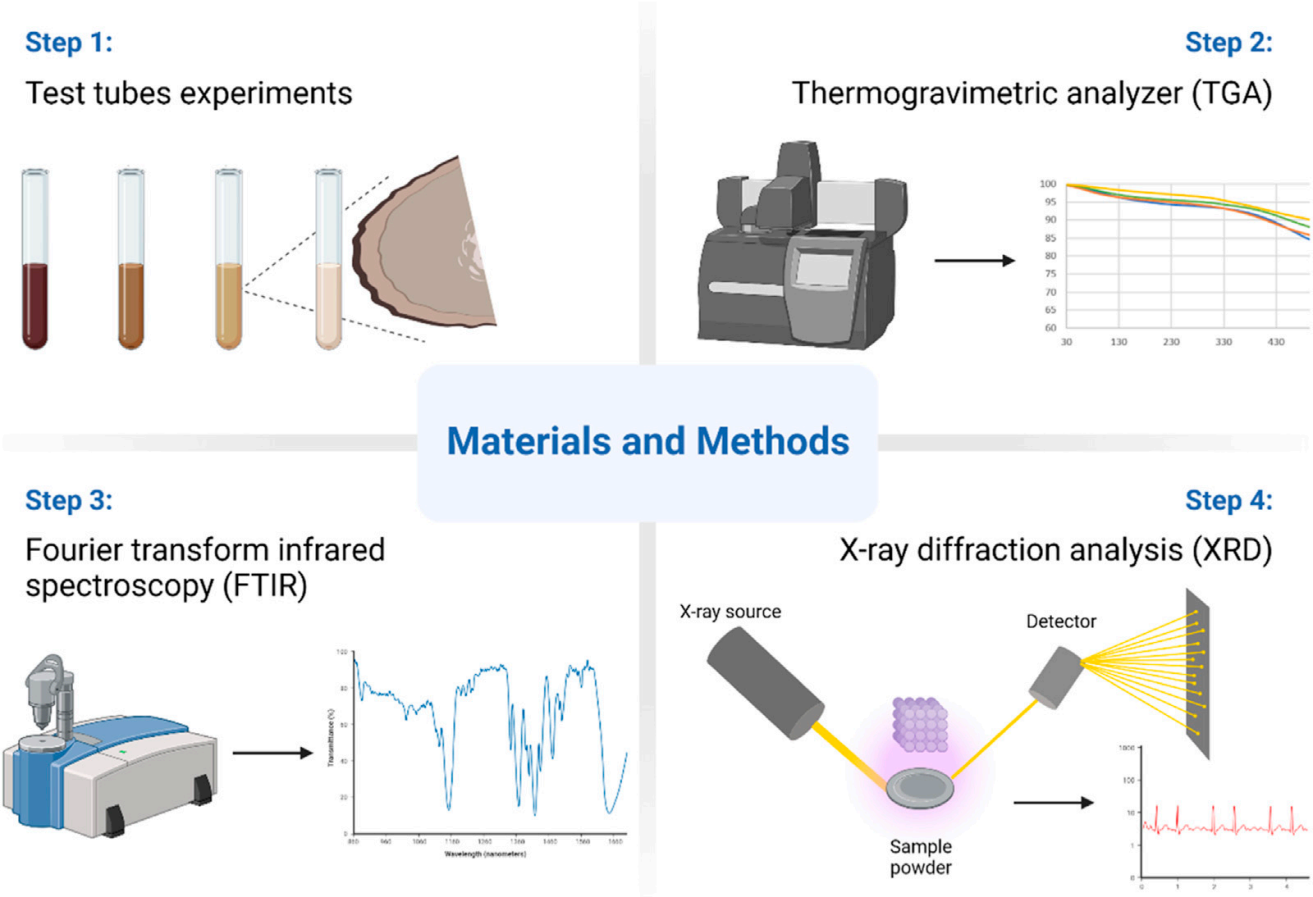
Research methodology.

The collected solids precipitates were analyzed by performing several analytical techniques including Thermogravimetric analysis (TGA), Fourier transform infrared spectroscopy (FTIR), and X-ray diffraction (XRD) for their composition and microstructure. The used instruments were Thermogravimetric analysis (TGA) by PerkinElmer^®^ TGA 8000, Fourier-transform infrared spectroscopy (FTIR) by Bruker INVENIO^®^ S FT-IR spectrometer, XRD from Panalytical Empyrean diffractometer. Figure 1 lists the methods and equipment used.

TGA is a thermal investigation technique that monitors the mass loss of material as temperature increases. In this study, TGA from Perkin Elmer (TGA8000) was employed to track the weight loss of a precipitated product as the temperature was raised to 900°C, with nitrogen gas flowing at a rate of 20 mL/min. The use of nitrogen gas created an inert environment that acted as an insulator to protect the sample from rapid combustion. During the TGA analysis, approximately 10 mg of the sample was accurately measured and loaded onto the TGA balance, and a temperature schedule was applied. The in-built software calculated the weight change in response to the temperature rise.

FTIR spectroscopy plays a primary role in material analysis, and it is applied to identify the functional groups and the composition of the precipitated product. FTIR involves exposing a sample to infrared radiation and plotting the amount of radiation absorbed at various wavelengths to produce an FTIR spectrum. The peaks and dips in the spectrum reveal the unique absorption patterns of each chemical bond and functional group in the sample to identify and distinguish between different substances and gain insights into their chemical composition.

XRD was applied to identify the product composition and compounds formed during precipitation. When a substance is exposed to X-rays and the resulting diffraction pattern is analyzed, valuable information about the arrangement of atoms within its crystal lattice can be obtained. This information is crucial for determining the precise crystal structure of the material. By comparing the diffraction pattern of a sample to a database of known patterns, we can identify the various compounds present in the sample with high accuracy.

## 3 Results and discussion

### 3.1 Test tube tests

**FIGURE 2:**
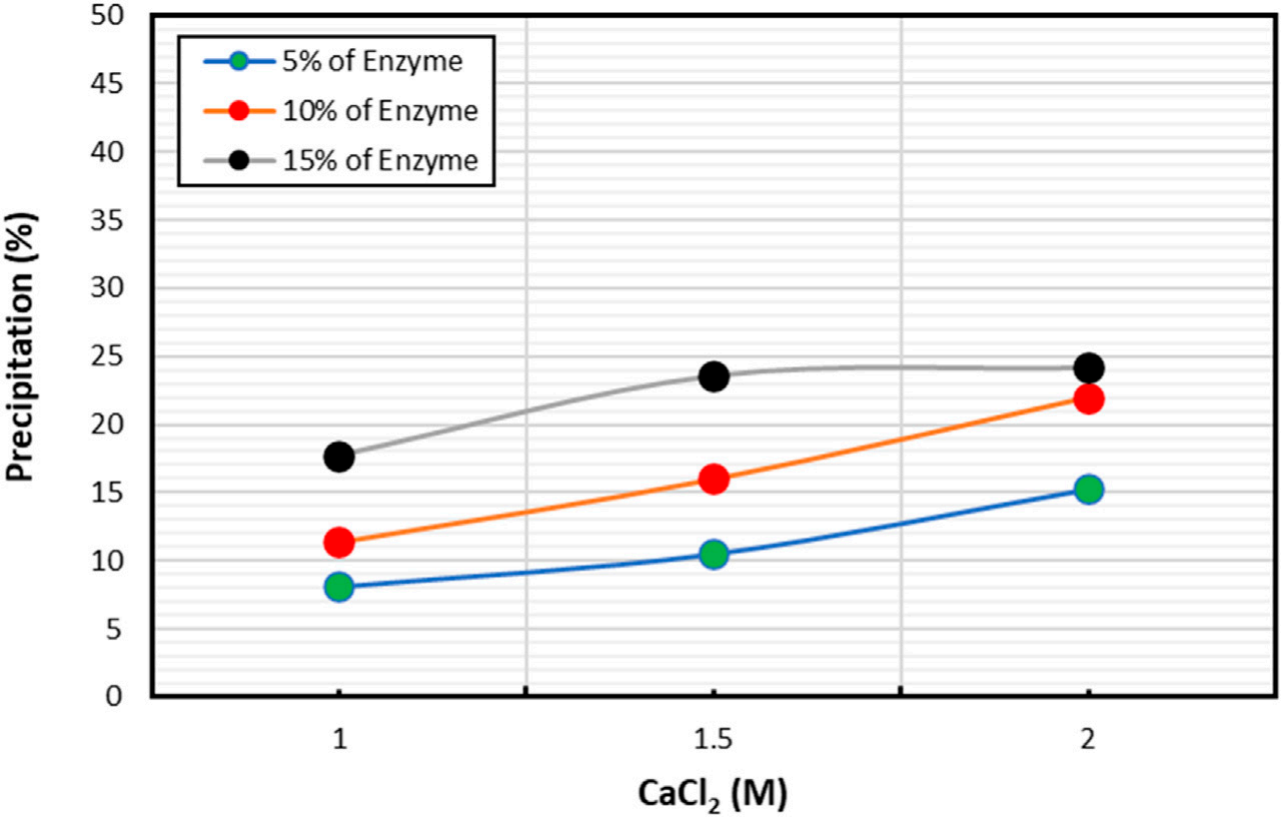
Evaluation of enzyme concentration sensitivity with CaCO_3_ precipitation (%).

Figure 2 illustrates the impact of enzyme concentration on precipitation product at varying molar concentrations of CaCl_2_. Specifically, the effect of CaCl_2_ was studied on three different enzyme concentrations (5%, 10%, and 15%), with CaCl2 concentration ranging from 1M to 2 M. Results indicated that higher enzyme concentration led to greater precipitation at a given CaCl_2_ molar concentration. As enzyme concentration increased, so did the amount of calcium carbonate precipitation, highlighting the positive relationship between enzyme concentration and precipitation.

Furthermore, the change in CaCl_2_ concentration at a particular enzyme concentration also influenced precipitation mass. In general, increasing the amount of CaCl_2_ led to higher precipitation percentages. However, at 15% enzyme concentration, a change in CaCl_2_ concentration from 1.5 M to 2 M did not result in significant changes. Conversely, lower enzyme concentrations exhibited a rising trend in precipitation with increasing CaCl_2_ concentration.

**FIGURE 3:**
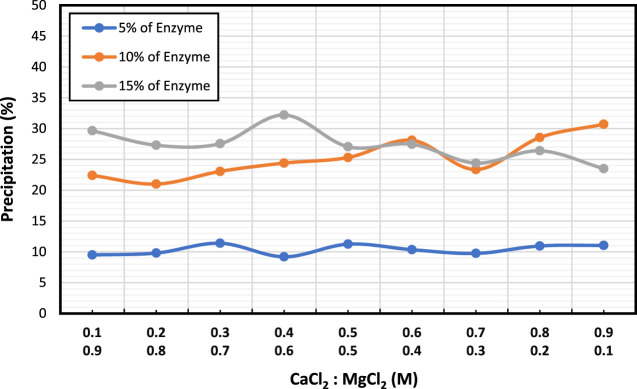
Evaluation of the synergistic effect of CaCl_2_ and MgCl_2_ on CaCO_3_ precipitation (%).

Figure 3 displays the combined effect of CaCl_2_ and MgCl_2_ at different molar concentrations on various enzyme concentrations (5%, 10%, and 15%). Results showed a similar trend to that of CaCl_2_, but with higher precipitations observed. As enzyme concentration increased, so did the amount of precipitation, except at the highest enzyme concentration, where a change in the CaCl_2_:MgCl_2_ molar ratio led to decreased precipitation. Notably, at 10% enzyme concentration, the trend was reversed, resulting in increased precipitation. At 5% enzyme concentration, precipitation remained at 10% and did not vary.

Overall, these results suggest that increasing enzyme concentration at a particular concentration of CaCl_2_ can lead to higher precipitation levels, with the highest precipitation obtained at 15% enzyme concentration.

The precipitation findings showed that 15% enzyme concentration yielded the highest CaCO_3_ precipitation and that (0.6:0.4) mol was the most effective molar ratio in terms of yielding the highest precipitation ratio therefore these concentrations are used for the subsequent tests. Adding MgCl_2_ to the reaction mixture improves the precipitation efficiency and increases the precipitation mass of CaCO_3_. Adding MgCl_2_ in EICP can lead to dolomite minerals forming, which have a higher precipitation mass than pure calcium carbonate (Putra et al., 2016; Putra et al., 2017a).

**FIGURE 4:**
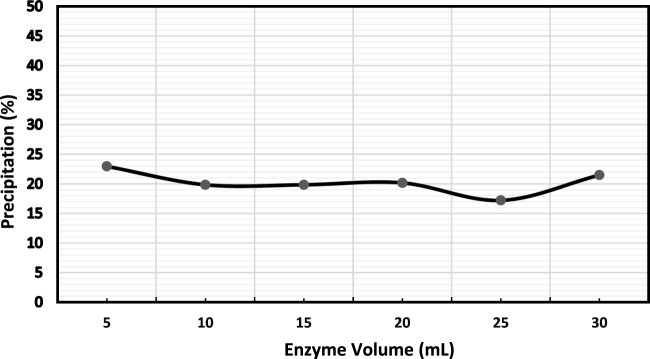
Evaluation of the enzyme volume sensitivity with CaCO_3_ precipitation (%).

Further, alpha-amylase’s volume to the EICP solution’s volume ratio was investigated. It was observed that adding more volume of enzymes did not have a discernible impact on precipitation as shown in Figure 4. Simply increasing enzyme volume may not change the precipitation as the solution is over-saturated with excessive enzymes with little or no substrate available. Hence, the excess enzymes may not play any role in the reaction (Ahenkorah et al., 2021a).

pH plays a crucial role in the precipitation of calcite in EICP induced by enzymes (Kim et al., 2018). The urease enzyme has an optimal pH range for activity, and the pH of the solution can impact the enzyme activity, which, in turn, can affect the rate of calcite precipitation. If the pH is too low, the enzyme may become denatured, and the activity may decrease, leading to a reduction in the rate of calcite precipitation. On the other hand, if the pH is too high, the concentration of carbonate ions may decrease, which could also limit calcite precipitation (Soon et al., 2014).

**FIGURE 5:**
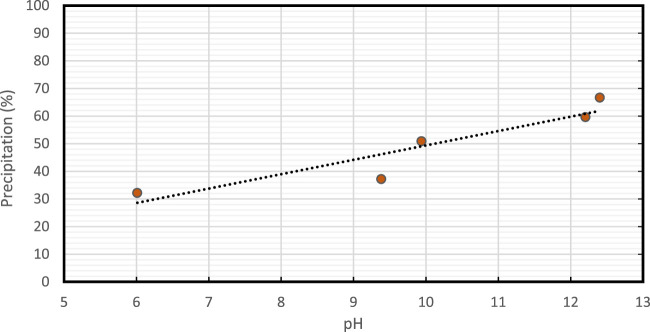
Effect of increasing pH on CaCO_3_ precipitation (%).

Therefore, it is important to maintain the pH of the solution within the optimal range to ensure efficient and effective calcite precipitation in EICP induced by enzymes. pH adjustments are often carried out using buffers, such as sodium bicarbonate, to maintain the desired pH range for the activity of the urease enzyme. The influence of solution pH on precipitation is illustrated in Figure 5. The amount of KOH used influenced the pH. There was a correlation between the increase in pH and the increase in precipitation (DeJong et al., 2010; Putra et al., 2017a; Kim et al., 2018). Our studies observed that precipitation % was increased with an increase in pH with 12.4 producing the highest amount of precipitation (65%).

**FIGURE 6:**
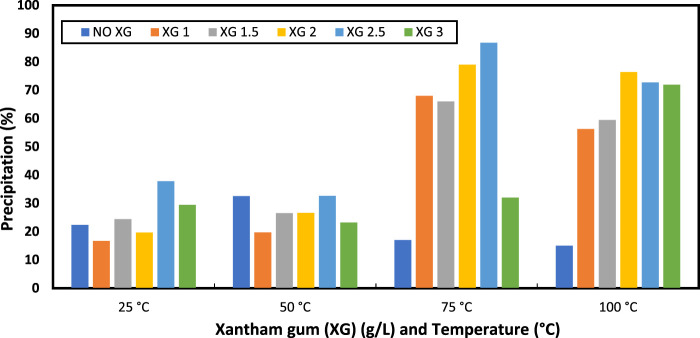
Evaluation of the effect of xanthan gum (XG) with temperature sensitivity on precipitation (%).

The effect of temperature and xanthan gum on precipitation is depicted in Figure 6. A total of four distinct temperatures (25, 50, 75, and 100°C) were used for the precipitation test. Five different concentrations of the xanthan gum (1, 1.5, 2, 2.5, and 3) g/L were evaluated on precipitation. The temperature was found to be affected the precipitation %. Precipitation varied with temperature in the base case. The difference was minimal from 25°C to 50°C. The difference was noticeable around 75°C. The precipitation dropped at 100°C, which may have been caused by the thermal degradation or inactiveness of the enzymes. Changes in precipitate concentration were brought about by adding more xanthan gum to the solution. With 2.5 g/L of xanthan gum, the maximum precipitation (87%) was produced at 75°C. The measured precipitation includes the dry polymer and other reaction products included.

### 3.2 Compositional and microstructural analysis

#### 3.2.1 Thermogravimetric analysis (TGA)

The TGA analysis was conducted on the precipitate to investigate their thermal stability. Eq. 3 is used to calculate the weight loss:

$$Weight\;loss=\frac{Current\;weight}{Initial\;weight}\times 100$$ (3)

**FIGURE 7:**
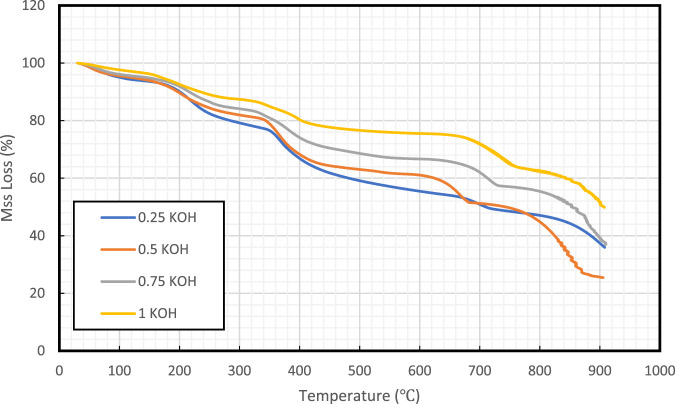
Thermal stability analysis plots for the samples vary in pH.

Figure 7 demonstrated the TGA of four different samples which differ in pH. The TGA analysis was conducted up to 900°C under an inert environment using N_2_. The samples lost their mass with an increase in temperature. The TGA analysis provides a step curve for all the samples. The mass loss varies with changes in temperature. The precipitates resulting from the high pH solution showed the highest thermal stability with approximately 50% weight loss at 900°C. The step curves showed the decomposition of samples with temperature. The mass loss occurred after 600°C showed the decomposition of CaCO_3_ into CaO and CO_2_ (Li et al., 2017). The decomposition before 600°C could be due to the decomposition of other reaction products formed during precipitation reaction. The mass loss below 200°C is due to water dehydration from the sample. The thermal stability of the precipitations showed that the reaction products obtained from all the solutions do not degrade at high temperature conditions of the reservoirs (<200°C). On other hand, the higher thermal stability was observed for the reaction products obtained from solution prepared with 1 M KOH which resulted in less mass loss compared to all other precipitated products.

#### 3.2.2 Fourier transform infrared spectroscopy (FTIR)

**FIGURE 8:**
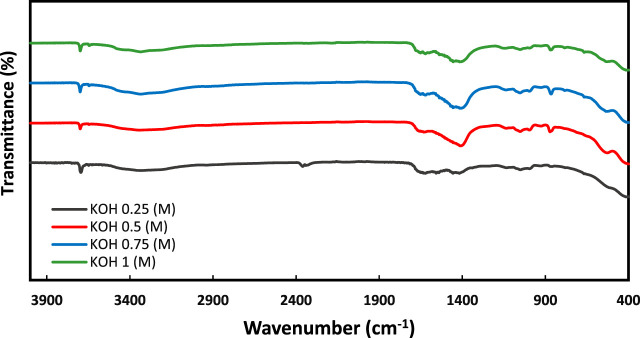
FTIR analysis for the effect of increasing pH with KOH.

FTIR (Fourier transform infrared spectroscopy) can be used to analyze the reaction product formed by EICP. It determines the chemical composition and functional groups present in the reaction product as Figure 8 shows the FTIR of reaction products resulting from precipitation tests performed on different pH solutions. In EICP, calcite or dolomite are formed by the reaction between Ca/Mg ions and carbonate ions produced from the hydrolysis of urea catalyzed by the urease enzyme. The FTIR spectrum of reaction products typically exhibits several characteristic absorption peaks. The most prominent peak is at around 1,400 cm^−1^, which corresponds to the asymmetric stretching vibration of the carbonate (CO_3_) group. Another peak is observed at around 870 cm^−1^, which corresponds to the bending vibration of the carbonate group. Additionally, the FTIR spectrum can also reveal the presence of other functional groups or impurities that may be present in the calcite product. In addition to these peaks, dolomite also exhibits peaks at around 1,040 cm^−1^, which correspond to the symmetric stretching and bending vibrations of the carbonate group. FTIR analysis can also be used to monitor the EICP process in real time. By analyzing the FTIR spectra of the reaction mixture at various stages of the process, it is possible to track the formation and growth of calcite crystals as well as the consumption of urea and the production of ammonium ions.

#### 3.2.3 X-ray diffraction (XRD)

The composition of the reaction products formed during an enzymatic reaction was determined by XRD analysis. The results showed that the sample containing urea 1 M, 0.6 M CaCl_2_, 0.4 M MgCl_2_, 15% enzyme, and 0.5 M KOH had the highest percentage (53.3%) of dolomite (CaMg(CO₃)₂ and sylvite (42.6%) with a minor concentration of calcite. On the other hand, the sample containing 1 M urea, 0.6 M CaCl_2_, 0.4 M MgCl_2_, 15% enzyme, and 1 M KOH had 29% of calcite (CaCO₃), 4.8% dolomite, 7.8% aragonite and 58.1% sylvite. The sample with 0.75 M KOH showed negligible amounts of both dolomite and calcite. Table 5 provides a detailed explanation of the different reaction products formed during the reaction.

**TABLE 5:** XRD analysis for the effect of increasing pH with KOH for a sample consisting of 1 M urea, 0.6 M CaCl_2_, 0.4 M MgCl_2_ and 15 wt% enzyme.

KOH [M]	Calcite [%]	Dolomite [%]	Aragonite [%]	Sylvite [%]
0.5	3	53.3	1.1	42.6
0.75	16.6	1.2	1.1	81.1
1	29.3	4.8	7.8	58.1

## 4 Proposed mechanism

**FIGURE 9:**
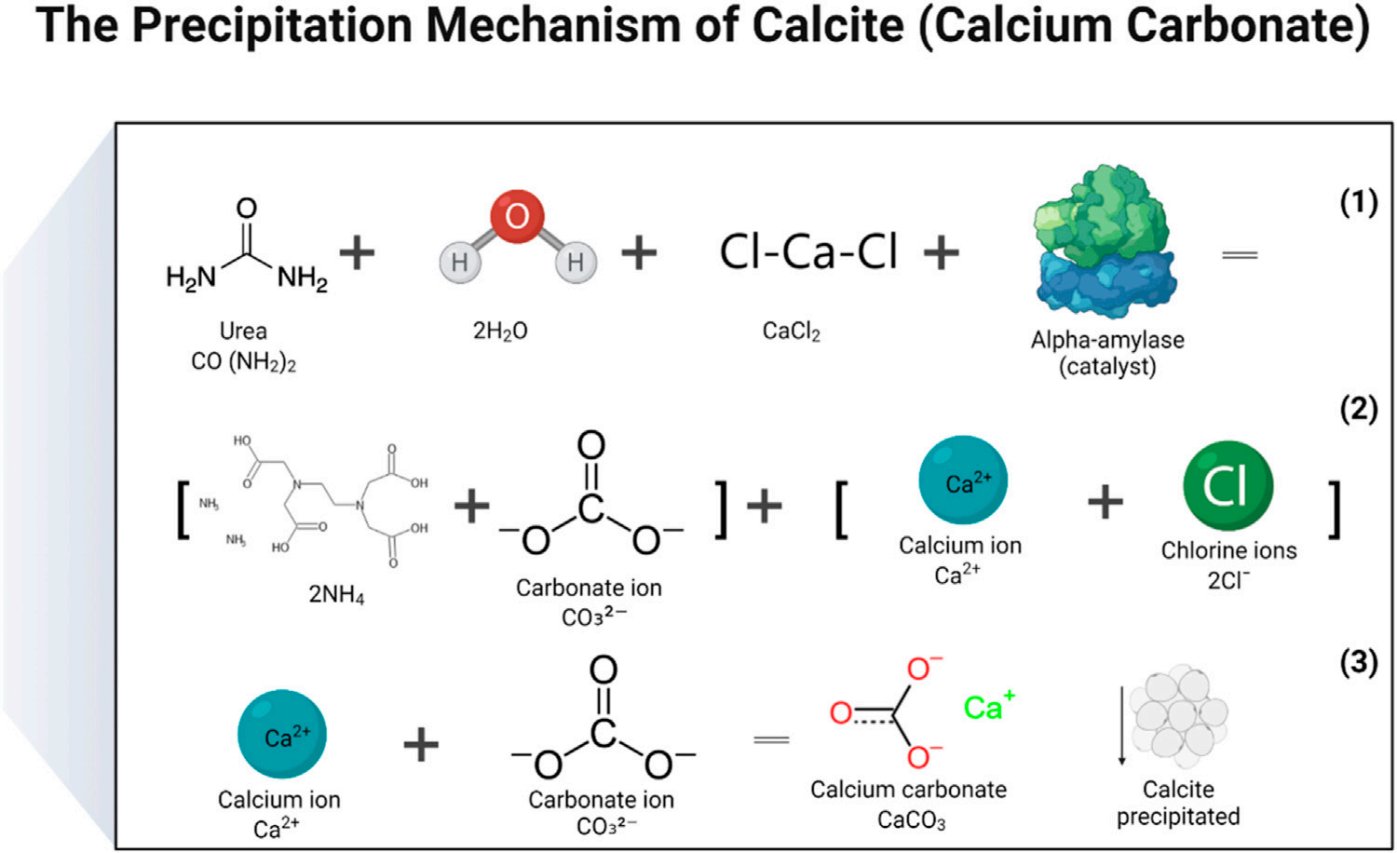
Reactions that have been carried out to produce a precipitate of calcium carbonate (calcite).

**FIGURE 10:**
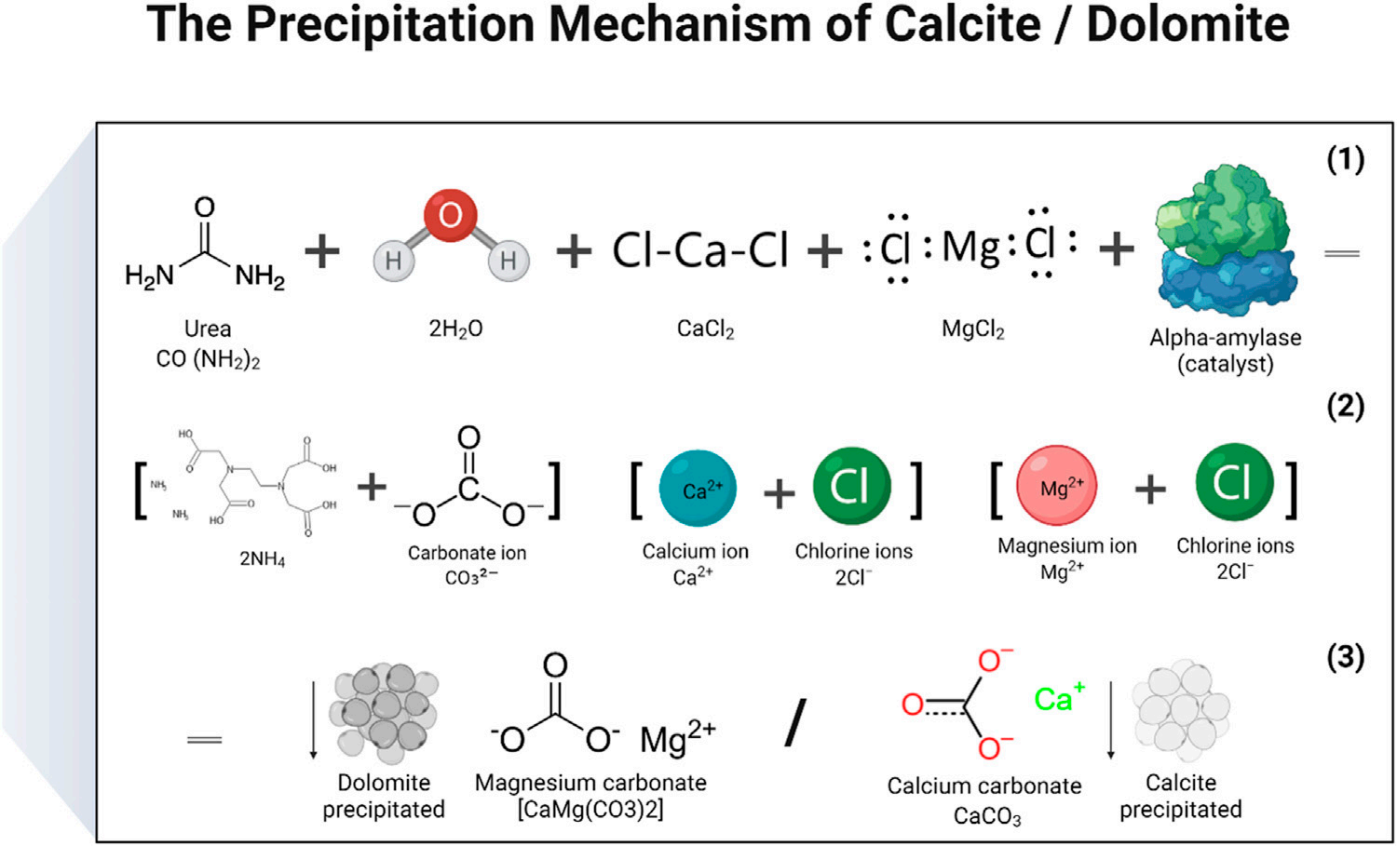
Reactions that have been carried out to produce a precipitate of calcium carbonate (calcite) and magnesium carbonate (dolomite).

In the study, two distinct mechanisms were proposed: 1. Calcite precipitation 2. Dolomite precipitation. To produce calcite or CaCO_3_, a solution of calcium chloride and urea was created by mixing the two substances, and then a catalytic agent, in the form of an enzyme, was added. During the process, the carbonate ions released from urea hydrolysis combined with the calcium ions that were released when CaCl_2_ dissociated in water, ultimately resulting in the formation of CaCO_3_. The enzymes served as a catalyst to facilitate the urea hydrolysis reaction. The basic chemical reactions that lead to the production of CaCO_3_ may be represented by the following (Eqs 4–6) (Putra et al., 2017b). The reactions responsible to produce calcite and dolomite are detailed in Figures 9, 10 (Tariq et al., 2022). There are some other reaction products formed during enzyme activity which were identified in XRD analysis. The products included aragonite and sylvite. Here only reactions were reported on the formation of calcite and dolomite.

$$CO{\left({NH}_{2}\right)}_{2}+2H_{2}O\to \;catalyst\;2{NH}_{4}++{CO}_{3}^{2-}$$ (4)

$${CaCl}_{2}\to {Ca}^{2+}+2{Cl}^{-}$$ (5)

$${Ca}^{2+}+{CO}_{3}^{2-}\to {CaCO}_{3}↓\left(precipitated\right)$$ (6)

The mechanism of calcite precipitation in Enzyme-Induced Calcite Precipitation (EICP) involves several steps (van Paassen et al., 2010; Putra et al., 2017b; Kong et al., 2019; Nayanthara et al., 2019; Ahenkorah et al., 2021b; Weinhardt et al., 2021):

1 Mixing of Urea and Salt: Mixing of CaCl_2_ saturated sand or rock with Urea.
2 Introduction of enzyme: Introduction of urease enzyme into the subsurface sand or rock saturated with urea and salt mix.
3 Hydrolysis of urea: Urease enzyme hydrolyzes urea, a naturally occurring nitrogen compound, into ammonium and carbonate ions.
4 Increase in pH: The hydrolysis reaction leads to an increase in the pH of the solution, as carbonate ions act as a base and react with water to form bicarbonate and hydroxide ions. The increase in pH enhances the solubility of calcium, which is required for the precipitation of calcite.
5 Precipitation of calcite: The carbonate ions produced by the hydrolysis of urea react with calcium ions present in the soil or rock, leading to the precipitation of calcite. Based on other metal ions presence, dolomite and other salt products formed during this reaction.
6 Formation of calcite crystals: The newly formed calcite crystals grow and aggregate, leading to the formation of a cement-like material that can fill void spaces and improve the strength and stiffness of the sand or rock.

Overall, the EICP process involves the hydrolysis of urea by microorganisms to produce carbonate ions, which react with calcium ions to form calcite. The process can be used for soil or sand stabilization, slope stabilization, and other applications, providing a sustainable and cost-effective alternative to traditional sand improvement techniques.

The predictable reactions to acquire precipitation of dolomite are given in (Eqs 7, 8).

$${MgCl}_{2}\to {Mg}^{2+}+{Cl}^{2-}$$ (7)

$${Mg}^{2+}+{CaCO}_{3}\left(s\right)\to Ca{\left({MgCO}_{3}\right)}_{2}\left(s\right)$$ (8)

## 5 Conclusion

In this study, the precipitation of calcium carbonate using alpha-amylase as an enzyme was investigated. Several experimental sensitivities were assessed, including enzyme concentration, enzyme volume, MgCl_2_ and xanthan gum effects, pH, and temperature. The results showed that a 15% enzyme concentration yielded the highest precipitate. The best molar ratio combination for CaCl_2_ and MgCl_2_ was (0.6:0.4) for the highest CaCO_3_ precipitation (32.2%). Enzyme volume in the solution did not significantly affect precipitation at a specific molar ratio. pH significantly affected precipitation; the precipitation yield increased with an increase in pH. The highest precipitate (87%) was obtained at 75°C with 2.5 g/L xanthan gum as a stabilizer. The temperature affects the enzyme activity and 75°C was found to be an effective temperature limit for the tested enzyme. TGA analysis revealed that the precipitated samples remained stable at temperatures and showed the reaction product was composed of CaCO_3_ and other complexes as decomposition occurred at 600°C. XRD analysis showed that the sample consisting of 1 M urea, 0.6 M CaCl_2_, 0.4 M MgCl_2_, 15 wt% enzyme, and 0.5 M KOH had the largest proportion of dolomite (53%). In contrast, the sample consisting of 1 M urea, 0.6 M CaCl_2_, 0.4 M MgCl_2_, 15 wt% enzyme, and 1 M KOH showed 29% calcite and 4.8% dolomite. The application of EICP in sand consolidation is increasing at a high pace. The limitation of this study is associated with the enzyme activity measurement, long-term and thermal stability, and utilization in producing sand pack samples.

## Data Availability

The original contributions presented in the study are included in the article/supplementary material, further inquiries can be directed to the corresponding authors.

## Abbreviations

CaO, calcium oxide; CO_2_, carbon dioxide; CaCl_2_, calcium chloride; EICP, enzyme induced calcite precipitation; FTIR, fourier transform infrared spectroscopy; KOH, potassium hydroxide; MICP, microbial induced calcite precipitation; MgCl_2_, magnesium chloride; TGA, thermogravimetric analysis; XRD, x-ray diffraction.

## References

[B1] Abduljabbar A. Mohyaldinn M. E. Younis O. Alghurabi A. Alakbari F. S. (2022). Erosion of sand screens by solid particles: A review of experimental investigations. J. Pet. Explor Prod. Technol. 12, 2329–2345. 10.1007/s13202-022-01467-4

[B2] Afrin H. (2017). A review on different types soil stabilization techniques. Int. J. Transp. Eng. Technol. 3, 19. 10.11648/j.ijtet.20170302.12

[B3] Ahenkorah I. Rahman M. M. Karim M. R. Beecham S. (2021a). Optimisation of chemical constituents on enzyme-induced carbonate precipitation in test-tube and soil. Geotechnical Res. 8, 66–84. 10.1680/jgere.21.00006

[B4] Ahenkorah I. Rahman M. M. Karim M. R. Beecham S. Saint C. (2021b). A review of enzyme induced carbonate precipitation (EICP): The role of enzyme kinetics. Sustain. Chem. 2, 92–114. 10.3390/suschem2010007

[B5] Alarifi S. A. Mustafa A. Omarov K. Baig A. R. Tariq Z. Mahmoud M. (2022). A review of enzyme-induced calcium carbonate precipitation applicability in the oil and gas industry. Front. Bioeng. Biotechnol. 10, 900881. 10.3389/fbioe.2022.900881 35795168 PMC9251129

[B6] Almajed A. Lateef M. A. Moghal A. A. B. Lemboye K. (2021). State-of-the-art review of the applicability and challenges of microbial-induced calcite precipitation (Micp) and enzyme-induced calcite precipitation (eicp) techniques for geotechnical and geoenvironmental applications. Cryst. (Basel) 11, 370. 10.3390/cryst11040370

[B7] Arukhe J. Uchendu C. Nwoke L. (2005). “Horizontal screen failures in unconsolidated, high-permeability sandstone reservoirs: Reversing the trend,” in Proceedings - SPE annual technical conference and exhibition. 10.2118/97299-ms

[B8] Banik N. Sarkar R. Uddin M. E. (2023). Assessment of strength and low-strain shear modulus of bio-cemented sand considering MICP treatment. Environ. Earth Sci. 82. 10.1007/s12665-023-10780-y 39757314

[B9] ben Mahmud H. Leong V. H. Lestariono Y. (2020). Sand production: A smart control framework for risk mitigation. Petroleum 6. 10.1016/j.petlm.2019.04.002

[B10] de Souza P. M. e Magalhães P. D. O. (2010). Application of microbial α-amylase in industry - a review. Braz. J. Microbiol. 41, 850–861. 10.1590/s1517-83822010000400004 24031565 PMC3769773

[B11] DeJong J. T. Mortensen B. M. Martinez B. C. Nelson D. C. (2010). Bio-mediated soil improvement. Ecol. Eng. 36, 197–210. 10.1016/j.ecoleng.2008.12.029

[B12] Farooq M. A. Ali S. Hassan A. Tahir H. M. Mumtaz S. Mumtaz S. (2021). Biosynthesis and industrial applications of α-amylase: A review. Arch. Microbiol. 203, 1281–1292. 10.1007/s00203-020-02128-y 33481073

[B13] Finnerty W. R. Singer M. E. (1983). Microbial enhancement of oil recovery. Bio/Technology 1, 47–54. 10.1038/nbt0383-47

[B14] Firoozi A. A. Guney Olgun C. Firoozi A. A. Baghini M. S. (2017). Fundamentals of soil stabilization. Int. J. Geo-Engineering 8, 26. 10.1186/s40703-017-0064-9

[B15] Ghadir P. Ranjbar N. (2018). Clayey soil stabilization using geopolymer and Portland cement. Constr. Build. Mater 188, 361–371. 10.1016/j.conbuildmat.2018.07.207

[B16] Gowthaman S. Nawarathna T. H. K. Nayanthara P. G. N. Nakashima K. Kawasaki S. (2021). “The amendments in typical microbial induced soil stabilization by low-grade chemicals, biopolymers and other additives: A review,” in Building materials for sustainable and ecological environment. 10.1007/978-981-16-1706-5_4

[B17] Hamdan N. Kavazanjian E. (2016). Enzyme-induced carbonate mineral precipitation for fugitive dust control. Geotechnique 66, 546–555. 10.1680/jgeot.15.P.168

[B18] Hu Q. Liu J. (2021). Production of α-amylase by Bacillus subtilis QM3 and its enzymatic properties. OAlib 08, 1–8. 10.4236/oalib.1107291

[B19] Hu W. Cheng W. C. Wen S. Yuan K. (2021). Revealing the enhancement and degradation mechanisms affecting the performance of carbonate precipitation in EICP process. Front. Bioeng. Biotechnol. 9, 750258. 10.3389/fbioe.2021.750258 34888301 PMC8650497

[B20] Ismail W. A. van Hamme J. D. Kilbane J. J. Gu J. D. (2017). Editorial: Petroleum microbial biotechnology: Challenges and prospects. Front. Microbiol. 8, 833. 10.3389/fmicb.2017.00833 28553269 PMC5427579

[B21] Kavazanjian E. Hamdan N. (2015). Enzyme induced carbonate precipitation (EICP) columns for ground improvement. 10.1061/9780784479087.209

[B22] Kim G. Kim J. Youn H. (2018). Effect of temperature, pH, and reaction duration on microbially induced calcite precipitation. Appl. Sci. Switz. 8, 1277. 10.3390/app8081277

[B23] Kong H. T. P. Kassim K. A. Umar M. Zango M. U. Muhammed A. S. Ahmad K. (2019). Microbially induced carbonate precipitations to improve residual soil at various temperatures. Bulletin of the Geological Society of Malaysia.

[B24] Lahiri D. Nag M. Sarkar T. Dutta B. Ray R. R. (2021). Antibiofilm activity of α-amylase from Bacillus subtilis and prediction of the optimized conditions for biofilm removal by response surface methodology (RSM) and artificial neural network (ANN). Appl. Biochem. Biotechnol. 193, 1853–1872. 10.1007/s12010-021-03509-9 33644831

[B25] Li X. G. Lv Y. Ma B. G. Wang W. Q. Jian S. W. (2017). Decomposition kinetic characteristics of calcium carbonate containing organic acids by TGA. Arabian J. Chem. 10, S2534–S2538. 10.1016/j.arabjc.2013.09.026

[B26] Mekonnen E. Kebede A. Tafesse T. Tafesse M. (2020). Application of microbial bioenzymes in soil stabilization. Int. J. Microbiol. 2020, 1–8. 10.1155/2020/1725482 PMC7424078 32831843

[B27] Molina-Fernández C. Luis P. (2021). Immobilization of carbonic anhydrase for CO2capture and its industrial implementation: A review. J. CO2 Util. 47, 101475. 10.1016/j.jcou.2021.101475

[B28] Nayanthara P. G. N. Dassanayake A. B. N. Nakashima K. Kawasaki S. (2019). Microbial Induced Carbonate Precipitation using a native inland bacterium for beach sand stabilization in nearshore areas. Appl. Sci. Switz. 9, 3201. 10.3390/app9153201

[B29] Noshi C. I. Schubert J. J. (2018). “Self-healing biocement and its potential applications in cementing and sand-consolidation jobs: A review targeted at the oil and gas industry,” in Society of petroleum engineers - SPE liquids-rich basins conference - north America 2018 (LRBC). 10.2118/191778-ms

[B30] Omoregie A. I. Palombo E. A. Nissom P. M. (2021). Bioprecipitation of calcium carbonate mediated by ureolysis: A review. Environ. Eng. Res. 26, 200379. 10.4491/eer.2020.379

[B31] Parker P. H. Treadway B. R. Brandt H. (1967). Oil well sand consolidation. I. Resins for a three‐step process. J. Appl. Polym. Sci. 11, 1667–1682. 10.1002/app.1967.070110907

[B32] Phillips A. J. Lauchnor E. Eldring J. Esposito R. Mitchell A. C. Gerlach R. (2013). Potential CO2 leakage reduction through biofilm-induced calcium carbonate precipitation. Environ. Sci. Technol. 47, 142–149. 10.1021/es301294q 22913538

[B33] Polowczyk I. Bastrzyk A. Fiedot M. (2016). Protein-mediated precipitation of calcium carbonate. Materials 9, 944. 10.3390/ma9110944 28774065 PMC5457223

[B34] Puppala A. J. Pedarla A. (2017). Innovative ground improvement techniques for expansive soils. Innov. Infrastruct. Solutions 2, 24. 10.1007/s41062-017-0079-2

[B35] Putra H. Yasuhara H. Kinoshita N. Hirata A. (2017b). Optimization of enzyme-mediated calcite precipitation as a soil-improvement technique: The effect of aragonite and gypsum on the mechanical properties of treated sand. Cryst. (Basel) 7, 59. 10.3390/cryst7020059

[B36] Putra H. Yasuhara H. Kinoshita N. Neupane D. Lu C. W. (2016). Effect of magnesium as substitute material in enzyme-mediated calcite precipitation for soil-improvement technique. Front. Bioeng. Biotechnol. 4, 37. 10.3389/fbioe.2016.00037 27200343 PMC4854898

[B37] Putra H. Yasuhara H. Kinoshita N. (2017a). Optimum condition for the application of enzyme-mediated calcite precipitation technique as soil improvement technique. Int. J. Adv. Sci. Eng. Inf. Technol. 7, 2145. 10.18517/ijaseit.7.6.3425

[B38] Rahim Z. Al-Malki B. Al-Kanaan A. (2010). “Selection of completion strategy for sand control and optimal production rate - field examples from Saudi Arabia’s ’Unayzah sandstone reservoir,” in Society of petroleum engineers - SPE asia pacific oil and gas conference and exhibition 2010 (APOGCE). 10.2118/131078-ms

[B39] Rahman M. M. Hora R. N. Ahenkorah I. Beecham S. Karim M. R. Iqbal A. (2020). State-of-the-art review of microbial-induced calcite precipitation and its sustainability in engineering applications. Sustain. Switz. 12, 6281. 10.3390/SU12156281

[B40] Raul D. Biswas T. Mukhopadhyay S. Kumar Das S. Gupta S. (2014). Production and partial purification of alpha amylase from bacillus subtilis (mtcc 121) using solid state fermentation. Biochem. Res. Int. 2014, 1–5. 10.1155/2014/568141 PMC3942096 24672727

[B41] Rodriguez-Navarro C. Cizer Ö. Kudłacz K. Ibañez-Velasco A. Ruiz-Agudo C. Elert K. (2019). The multiple roles of carbonic anhydrase in calcium carbonate mineralization. CrystEngComm 21, 7407–7423. 10.1039/c9ce01544b

[B42] Saghandali F. Baghban Salehi M. Hosseinzadehsemnani R. Moghanloo R. G. Taghikhani V. (2022). A review on chemical sand production control techniques in oil reservoirs. Energy & Fuels 36, 5185–5208. 10.1021/acs.energyfuels.2c00700

[B43] Saif A. Cuccurullo A. Gallipoli D. Perlot C. Bruno A. W. (2022). Advances in enzyme induced carbonate precipitation and application to soil improvement: A review. Materials 15, 950. 10.3390/ma15030950 35160900 PMC8840754

[B44] Sharma M. Satyam N. (2023). “Influence of freezing–thawing cycles on biotreated sand using MICP,” in Lecture notes in civil engineering (Springer Science and Business Media Deutschland GmbH), 383–389. 10.1007/978-981-19-6774-0_37

[B45] Sharma M. Satyam N. Reddy K. R. (2022). Liquefaction resistance of biotreated sand before and after exposing to weathering conditions. Indian Geotechnical J. 52, 328–340. 10.1007/s40098-021-00576-x

[B46] Sharma M. Satyam N. Reddy K. R. (2021). Rock-like behavior of biocemented sand treated under non-sterile environment and various treatment conditions. J. Rock Mech. Geotechnical Eng. 13, 705–716. 10.1016/j.jrmge.2020.11.006

[B47] Soon N. W. Lee L. M. Khun T. C. Ling H. S. (2014). Factors affecting improvement in engineering properties of residual soil through microbial-induced calcite precipitation. J. Geotechnical Geoenvironmental Eng. 140. 10.1061/(asce)gt.1943-5606.0001089

[B48] Stein N. Odeh A. S. Jones L. G. (1974). Estimating maximum sand-free production rates from friable sands for different well completion geometries. JPT, J. Petroleum Technol. 26, 1156–1158. 10.2118/4534-PA

[B49] Tariq Z. Mahmoud M. Alahmari M. Bataweel M. Mohsen A. (2022). Lost circulation mitigation using modified enzyme induced calcite precipitation technique. J. Pet. Sci. Eng. 210, 110043. 10.1016/j.petrol.2021.110043

[B50] Umar M. Kassim K. A. Ping Chiet K. T. (2016). Biological process of soil improvement in civil engineering: A review. J. Rock Mech. Geotechnical Eng. 8, 767–774. 10.1016/j.jrmge.2016.02.004

[B51] van Paassen L. A. Daza C. M. Staal M. Sorokin D. Y. van der Zon W. van Loosdrecht M. C. M. (2010). Potential soil reinforcement by biological denitrification. Ecol. Eng. 36, 168–175. 10.1016/j.ecoleng.2009.03.026

[B52] Verma A. S. Agrahari S. Rastogi S. Singh A. (2011). Biotechnology in the realm of history. J. Pharm. Bioallied Sci. 3, 321. 10.4103/0975-7406.84430 21966150 PMC3178936

[B53] Wang L. Cheng W.-C. Xue Z.-F. Xie Y.-X. Lv X.-J. (2023). Feasibility study of applying electrokinetic technology coupled with enzyme-induced carbonate precipitation treatment to Cu- and Pb-contaminated loess remediation. J. Clean. Prod. 401, 136734. 10.1016/j.jclepro.2023.136734

[B54] Wang L. Cheng W. C. Xue Z. F. (2022). Investigating microscale structural characteristics and resultant macroscale mechanical properties of loess exposed to alkaline and saline environments. Bull. Eng. Geol. Environ. 81, 146. 10.1007/s10064-022-02640-z

[B55] Weinhardt F. Class H. Vahid Dastjerdi S. Karadimitriou N. Lee D. Steeb H. (2021). Experimental methods and imaging for enzymatically induced calcite precipitation in a microfluidic cell. Water Resour. Res. 57. 10.1029/2020WR029361

[B56] Weirich J. Li J. Abdelfattah T. Pedroso C. (2013). Frac packing: Best practices and lessons learned from more than 600 operations. Available at: http://onepetro.org/DC/article-pdf/28/02/119/2093482/spe-147419-pa.pdf/1 .

[B57] Winoto V. Keawsawasvong S. (2022). Response surface methodology for optimizing stabilization of clay soils using bacterial calcium carbonate precipitation. Transp. Infrastruct. Geotechnol. 9, 890–898. 10.1007/s40515-021-00205-3

[B58] Xu G. Tang Y. Lian J. Yan Y. Fu D. (2017). Mineralization process of biocemented sand and impact of bacteria and calcium ions concentrations on crystal morphology. Adv. Mater. Sci. Eng. 2017, 1–13. 10.1155/2017/5301385

[B59] Xue Z.-F. Cheng W.-C. Wang L. Song G. (2021). Improvement of the shearing behaviour of loess using recycled straw fiber reinforcement. KSCE J. Civ. Eng. 25 (9), 3319–3335. 10.1007/s12205-021-2263-3

[B60] Xue Z.-F. Cheng W.-C. Xie Y.-X. Wang L. Hu W. Zhang B. (2023). Investigating immobilization efficiency of PB in solution and loess soil using bio-inspired carbonate precipitation. Environ. Pollut. 322, 121218. 10.1016/j.envpol.2023.121218 36764377

[B61] Yuan P. B. Dai P. F. Chen W. W. (2016). “Enzyme induced calcite precipitation (EICP) to strengthen the ability of anti-wind erosion of earthen archaeological sites,” in Ancient underground opening and preservation - proceedings of the international symposium on scientific problems and long-term preservation of large-scale (Ancient Underground Engineering). 10.1201/b19169-47

[B62] Zamani A. Montoya B. M. Gabr M. A. (2019). Investigating challenges of *in situ* delivery of microbial-induced calcium carbonate precipitation (MICP) in fine-grain sands and silty sand. Can. Geotechnical J. 56, 1889–1900. 10.1139/cgj-2018-0551

[B63] Zamani A. Xiao P. DeJong J. T. Boulanger R. W. Wilson D. W. Carey T. J. (2021). MICP treatment to mitigate soil liquefaction-induced building settlements. 10.1061/9780784483411.030

